# Effect of Organic Selenium on the Homeostasis of Trace Elements, Lipid Peroxidation, and mRNA Expression of Antioxidant Proteins in Mouse Organs

**DOI:** 10.3390/ijms24119704

**Published:** 2023-06-02

**Authors:** Inga Staneviciene, Dovydas Levinas, Ilona Sadauskiene, Arunas Liekis, Dale Viezeliene, Lolita Kursvietiene, Rima Naginiene, Dale Baranauskiene, Vaida Simakauskiene, Paulina Vaitkiene, Giedre Miniotaite, Jurgita Sulinskiene

**Affiliations:** 1 Department of Biochemistry, Medical Academy, Lithuanian University of Health Sciences, A. Mickeviciaus St. 9, LT-44307 Kaunas, Lithuania; 2 Neuroscience Institute, Lithuanian University of Health Sciences, Eiveniu St. 4, LT-50009 Kaunas, Lithuania

**Keywords:** selenium, iron, zinc, copper, lipid peroxidation, catalase, selenoprotein P, superoxide dismutase, mRNA expression

## Abstract

(1) In this study we determined the effect of long-term selenomethionine administration on the oxidative stress level and changes in antioxidant protein/enzyme activity; mRNA expression; and the levels of iron, zinc, and copper. (2) Experiments were performed on 4–6-week-old BALB/c mice, which were given selenomethionine (0.4 mg Se/kg b.w.) solution for 8 weeks. The element concentration was determined via inductively coupled plasma mass spectrometry. mRNA expression of *SelenoP*, *Cat*, and *Sod1* was quantified using real-time quantitative reverse transcription. Malondialdehyde content and catalase activity were determined spectrophotometrically. (3) After long-term SeMet administration, the amount of Se increased by 12-fold in mouse blood, 15-fold in the liver, and 42-fold in the brain, as compared to that in the control. Exposure to SeMet decreased amounts of Fe and Cu in blood, but increased Fe and Zn levels in the liver and increased the levels of all examined elements in the brain. Se increased malondialdehyde content in the blood and brain but decreased it in liver. SeMet administration increased the mRNA expression of selenoprotein P, dismutase, and catalase, but decreased catalase activity in brain and liver. (4) Eight-week-long selenomethionine consumption elevated Se levels in the blood, liver, and especially in the brain and disturbed the homeostasis of Fe, Zn, and Cu. Moreover, Se induced lipid peroxidation in the blood and brain, but not in the liver. In response to SeMet exposure, significant up-regulation of the mRNA expression of catalase, superoxide dismutase 1, and selenoprotein P in the brain, and especially in the liver, was determined.

## 1. Introduction

Selenium (Se) is one of the trace minerals that is required to maintain various functions of the body [1]. Human beings obtain Se from the food or its supplements as both organic (e.g., selenomethionine (SeMet), selenocysteine (Sec)) and inorganic (e.g., selenites, selenates) origins and absorb about 80–95 percent of it [2,3]. The biological significance of Se is based on selenoproteins, the structure of which contains the unique amino acid selenocysteine [4]. This amino acid, also called the twenty-first amino acid, is an analog of cysteine with selenium instead of sulfur. The human selenoproteome is encoded by 25 genes [5], while in rodents, it is encoded by 24 genes [6]. According to the functions they perform, human selenoproteins are divided into subfamilies, such as those related to antioxidant activity (*GPX1*, *GPX2*, *GPX3*, *GPX4* genes), regulation of the redox state (*TXNRD1*, *TXNRD2*, *TXNRD3*, *MSRB1*, *SELENOH*, *SELENOM*, *SELENOW* genes), thyroid hormone metabolism (*DIO2*, *DIO3* genes), Se transport and storage (*SELENOP* gene), calcium metabolism (*SELENOK*, *SELENOT* genes), selenophosphate synthesis *(SEPHS2* gene), protein folding (*SELENOF*, *SELENOI*, *SELENOS* genes), myogenesis (*SELENON* gene), and protein AMPylation (*SELENOO* gene) [7,8,9], while the functions of SELENOV and GPX6 are still not clear [10].

According to the World Health Organization, the recommended daily dose of Se for adults is set at 55–70 µg/day [1]. The intake of Se must be strictly regulated as the ranges of insufficient intake and excess, which has a toxic effect, are quite narrow [11]. The liver plays a key role in selenium homeostasis in the body. The content of available Se determines the different expression levels of different selenoproteins, which depend on both the species and the organ. When the content of Se is insufficient, it is primarily used for the synthesis of more key vital selenoproteins (e.g., glutathione peroxidase, selenoprotein P), thus creating a hierarchy of them [3]. This hierarchy ensures the appropriate distribution of Se in the tissues and primarily provides it to the priority organs, such as the brain and endocrine glands [12,13].

Selenoprotein P, located on chromosome 5q31 and encoded by the *SELENOP* gene, is the main selenoprotein in blood plasma, which accounts for about 50% of total blood plasma Se [14]. Selenoprotein P, as a biomarker to assess Se uptake and content in the body, has been the recent focus of researchers [15,16,17]. As compared with other selenoproteins, selenoprotein P is special in that it has ten Sec residues in its structure, while many other selenoproteins have only one or two [18]. The abundance of Sec residues determines the ability of this protein to perform many different functions. [19]. Selenoprotein P is not only responsible for selenium distribution in the body, but is also known to possess antioxidant, glutathione peroxidase-like activity, which contributes to decreased lipid peroxidation, peroxynitrite, and metal scavenging activities [20,21,22,23]. This protein is mainly synthesized in the liver, and from there, it is transported to extrahepatic tissues, including the brain [3]; however, in vitro studies have shown that *SELENOP* gene expression also occurs in neurons, astrocytes, testicular Leydig cells, adipocytes, and pancreatic β-cells [12,24]. If Se concentrations exceed the body’s optimal amount, some of the Se is methylated and glycosylated and excreted in the urine. When the amount of Se in the body is insufficient, the synthesis of intracellular selenoproteins decreases and the formation of excretable Se forms stops; however, the synthesis of selenoprotein P is activated [3]. Therefore, the amount of available Se and thus changes in the expression of selenoproteins are related to the development of nervous system diseases (Alzheimer’s, epilepsy, Parkinson’s, and schizophrenia) and kidney, liver, autoimmune, and other type of illnesses [12,19,25,26,27,28].

At supranutritional doses, organic SeMet, as well as inorganic Se, is known to exhibit prooxidant activity and thus be toxic. The pro-oxidant activity of SeMet is based on the oxidation of thiol groups in proteins, the depletion of reduced glutathione, and the generation of free radicals and reactive oxygen species (ROS), all leading to oxidative stress, a harmful process that causes damage to various cellular structures, tissues, and organs [29]. Free radicals can directly affect lipids, causing their peroxidation [30]. There are data suggesting that the pro-oxidative activity of Se may increase the risk of brain or endocrine system disorders and increase the probability of cancer [31,32].

The scientific data provide rather contradictory information about what Se concentrations should be considered as physiological in the brains of humans or experimental animals. This lack of information is critical, since restoring or manipulating micronutrient levels in the brain is one of the emerging treatment strategies for neurodegenerative and other diseases [33]. There is also very little data on how Se deficiency and especially excess can affect the homeostasis of certain trace elements, such as iron (Fe), zinc (Zn), and copper (Cu), in the brain. After all, these trace elements, being cofactors that ensure enzymatic activity, are important for synapse plasticity, neuronal migration and differentiation, the modulation of nerve impulse propagation, the myelination of axons, or the regulation of neurogenesis [34,35,36,37,38,39]. Zn and Cu are necessary for antioxidant nervous tissue protection [40,41], while their imbalance is associated with the onset and/or progression of neurodegenerative disorders [42,43,44,45,46]. The liver plays a central role in Zn and Cu homeostasis; it not only accumulates these metals, but also synthesizes various proteins or regulatory molecules (protein receptors, carriers, transcription factors) involved in their metabolism. Thus, on the one hand, the liver maintains metal homeostasis, and on the other hand, a metal imbalance affects the normal functioning of the liver.

Various research confirms that organic compounds of Se are better absorbed and accumulated in the tissue and have lower toxicity and higher antioxidant capacity compared to inorganic ones [29,47,48,49]; to achieve an antioxidant effect, the daily dose of Se must be several times higher than the recommended one [50]. However, there is still insufficient amount of scientific data in order to effectively and safely use Se for both preventive and therapeutic purposes, while the information that does exist is quite contradictory, due the existing bimodal action of selenium. We know already that the human selenoproteome is encoded by 25 selenoprotein genes and is highly regulated by Se bioavailability and a tissue-specific hierarchy; however, little is known about the effect of Se on the expression of genes encoding non-selenium proteins [51].

Therefore, the aim of this study was to determine how the long-term (8 weeks) administration of organic selenomethionine (0.4 mg Se/kg body weight) affects the level of oxidative stress in different laboratory mouse tissues and to evaluate the effect of Se on the mRNA expression of selenoprotein P, as well as the mRNA expression of selenium-free antioxidant system enzymes—superoxide dismutase (SOD) and catalase (CAT). We also sought to evaluate how an excess of SeMet affects the homeostasis of biogenic elements, such as Fe, Cu and Zn, thus at least partially filling the data gap in the scientific data.

## 2. Results

### 2.1. Evaluation of Selenium Effect on Body Weight and Relative Organ Mass Index of Mice

To assess the overall systemic effects of SeMet *per os* administration on body weight and the relative organ mass index, each laboratory BALB/c mouse was weighed once a week throughout the study period; tissue weights of the brain, liver, and kidney were determined at the end of the experiment.

**Figure 1:**
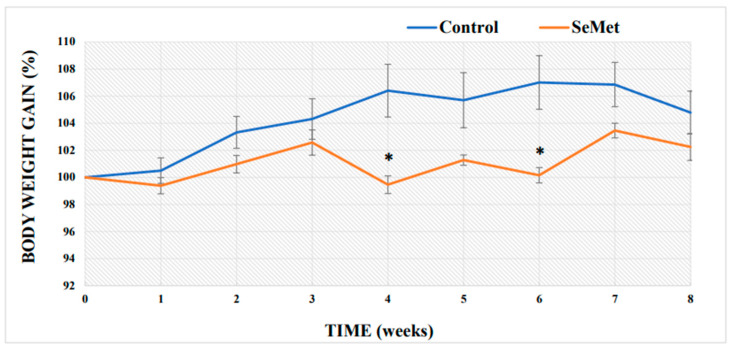
Time course of body weight gain of the control-group mice and the mice orally treated with selenomethionine (SeMet) solution for 8 weeks. The medium weight gain in the groups was expressed as a percentage, and the initial weights of the mice in each group were equated to 100%. The model of selenium exposure to mice is described in the Methods section. The data were obtained by measuring the body weights of 16 mice in each group. *—differences are statistically significant in comparison to the control group; *p* ˂ 0.05.

**Figure 2:**
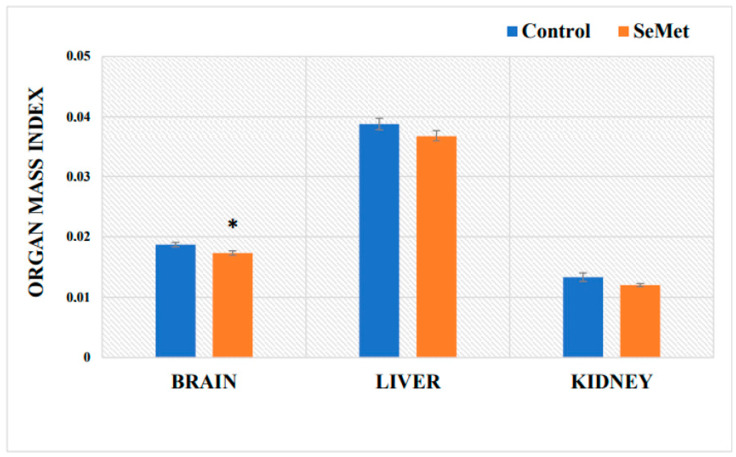
Relative mass index of the mouse brain, liver, and kidney (g of organ mass/g of body weight) of control-group mice and mice orally treated with selenomethionine (SeMet) solution for 8 weeks. The model of selenium exposure to mice is described in the Methods section. The data were obtained by measuring the body weights and organ masses of 16 mice in each group. Results were expressed as the mean ± SEM. *—differences are statistically significant in comparison to the control group; *p* ˂ 0.05.

In toxicological studies, body mass and relative organ mass are considered significant toxicity assessment criteria that reflect the general condition of the organism [52]. An evaluation of the changes in the body mass of the control mice showed a steady increase during the first four weeks of the experiment; at the fifth week, the body weight growth slightly slowed down and then remained at a similar level until the end of the experiment (Figure 1). Body weight analysis of the experimental group of mice showed that the weights of the mice exposed to SeMet were lower than those of the control mice throughout the experiment. Compared to the body weight gain of the control mice, the body weight gain of the Se-treated mice was statistically significantly lower by 6.9% in the first four weeks of the study and by 6.8% lower in the fifth–sixth weeks of the study (*p* < 0.05). An evaluation of a final weight gain of the mice showed that the weight of the mice treated with SeMet increased by 2.2% as compared to the initial weight, while that of the mice in the control group increased by 4.8%; thus, one can say that the growth of SeMet-treated mice was just slightly slower (by 2.6%) compared to the growth of control mice; however, the difference was not statistically significant (Figure 1). The relative mouse organ mass index (brain, liver, and kidney) determined after 8 weeks of exposure to SeMet showed a statistically significant reduction (by 7.5%) in the relative brain mass index as compared to that in the control mouse group; liver and kidney mass indices of organic Se-treated mice also decreased; however, the differences as compared to controls, were not statistically significant (Figure 2).

### 2.2. Evaluation of Selenium, Iron, Zinc, and Copper Concentrations in Mouse Tissues

At this stage of the study, we determined Se concentrations in the blood, brain, and liver of experimental mice after 8 week-long SeMet-enriched water consumption and compared them with those in the control group of mice (Table 1). At the end of the experiment, the concentration of Se in the blood of control mice was 210.508 ± 24.14 µg/L, while the Se blood concentration of SeMet-treated mice was found to be statistically significantly increased by 1100% (2526.303 ± 181.06 µg/L) as compared to that in the controls; Se content here was 12-fold higher than that of the control group. The concentration of Se in the liver of control mice reached 0.615 ± 0.095 µg/g; meanwhile, the livers of SeMet-treated mice accumulated 9.490 ± 0.33 µg/g of selenium, which was increased by 1443% or 15.4-fold (*p* < 0.05) higher than that in the livers of control mice. The average amount of Se in the brains of control mice was 0.061 ± 0.012 µg/g. The amount of Se, which accumulated in the brains of mice due to long-term SeMet administration, was 2.573 ± 0.147 µg/g and that was 42 times (by 4118%) higher than the amount of selenium found in the brains of control mice (*p* ˂ 0.05).

There are many data available on the physiological concentrations of biogenic elements in human and animal tissues; however, the data about the effect of Se on the homeostasis of other elements are very sparce. We sought to evaluate the effect of Se on the homeostasis of some key trace elements, such as iron, copper, and zinc, which are also cofactors of some enzymes of the antioxidant system.

The concentrations of Fe, Cu, and Zn in the blood of laboratory mice, presented in Table 2, demonstrate a significant reduction in the Fe content (by 30%) in the blood of SeMet-treated mice. The concentration of Fe in SeMet-administered mice was 497.121 ± 9.85 mg/L, while the Fe level in the blood of control mice was 706.097 ± 32.35 mg/L. The amount of Zn in the blood of mice exposed to SeMet remained almost at the control level (6.640 ± 0.29 mg/L). The slight decrease in the content of Zn (6.307 ± 0.22 mg/L), compared to that in the controls, was only 5% although it was not statistically significant. The concentration of Cu in the blood of control mice was 0.985 ± 0.064 mg/L, while the content of Cu in the blood of SeMet-administrated mice was significantly (by 40%) lower than that of control mice and only reached 0.594 ± 0.026 mg/L.

It was found that the concentration of Fe in the livers of SeMet-administered mice reached 272.894 ± 9.52 µg/g, and that was 2.3-fold (by 129%) higher as compared to the control level (119.068 ± 11.26 µg/g) (Table 1). The concentration of Cu in the liver of mice that consumed SeMet was 6.877 ± 0.12 µg/g − 11.8% lower compared to that of control (7.795 ± 0.42 µg/g) mice, and this decrease was statistically significant. The concentration of Zn in the livers of control-group mice was 32.453 ±1.40 µg/g, while in the liver of mice treated with SeMet solution, the content of Zn was 61% higher and reached 52.213 ± 0.99 µg/g (*p* < 0.05) (Table 2).

As can be seen from the results presented in Table 2 that the content of Fe in the brain of the SeMet solution-exposed mouse group reached 38.803 ± 2.16 µg/g and was 1.4 times (by 42%) higher as compared to that in the control group (27.265 ± 1.9 µg/g). The amount of Cu (6.266 ± 0.58 µg/g) accumulated in the long-term SeMet-treated mouse brain was statistically significantly higher, by 68%, compared to that in the control group (3.715 ± 0.16 µg/g). Long-term SeMet consumption also increased zinc levels in the brain; in SeMet-treated mouse brains, the amount of Zn was 24.624 ± 2.39 µg/g, while in control mice, the content of Zn was 16.863 ± 0.51 µg/g, which was significantly (by 46%) lower compared to that in SeMet-treated mouse brains.

### 2.3. Evaluation of Malondialdehyde Content in Mouse Tissues after Exposure to Selenomethionine

In order to find out whether the high amounts of Se, accumulated in the tissues due to SeMet consumption, have a pro-oxidant effect and can induce cell oxidative stress, we sought to evaluate the level of polyunsaturated fatty acid peroxidation in our examined tissue. The formation of malondialdehyde (MDA)—the final product of lipid peroxidation—was chosen as a marker for this purpose.

**Figure 3:**
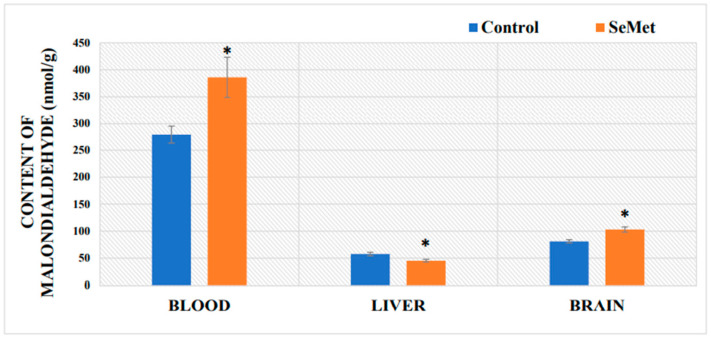
Content of malondialdehyde in mouse brains and livers of control-group mice and mice orally treated with selenomethionine (SeMet) solution for 8 weeks. The model of selenium exposure to mice is described in the Methods section. Data represents the results of 16 separate experiments (8 mice in each group). Results were expressed as the mean ± SEM. *—differences are statistically significant in comparison to the control group; *p* ˂ 0.05.

The results of MDA formation in the blood and tissue of mice after 8 weeks of SeMet consumption are presented in Figure 3. Our results showed that the concentration of MDA in the blood of mice exposed to SeMet was 386.00 ± 37.23 µmol/L and was significantly higher (by 34%) as compared to MDA levels in the blood of control mice (287.74 ± 15.64 µmol/L) (*p* < 0.05). SeMet exposure also seemed to increase lipid peroxidation in the brain, as the MDA concentration there reached 102.98 ± 4.8 nmol/g and was statistically significantly higher (by 27%) as compared to the MDA in the control (81.13 ± 3.06 nmol/g). The opposite effect of Se was observed in the liver; the content of MDA in SeMet-exposed mouse livers was 45.39 ± 2.48 nmol/g and that was by 21% lower, as compared to MDA levels in the liver of the control group of mice (57.59 ± 3.32 nmol/g) (Figure 3).

### 2.4. Determination of Mouse Brain and Liver Catalase Activity after Exposure to Selenomethionine

**Figure 4:**
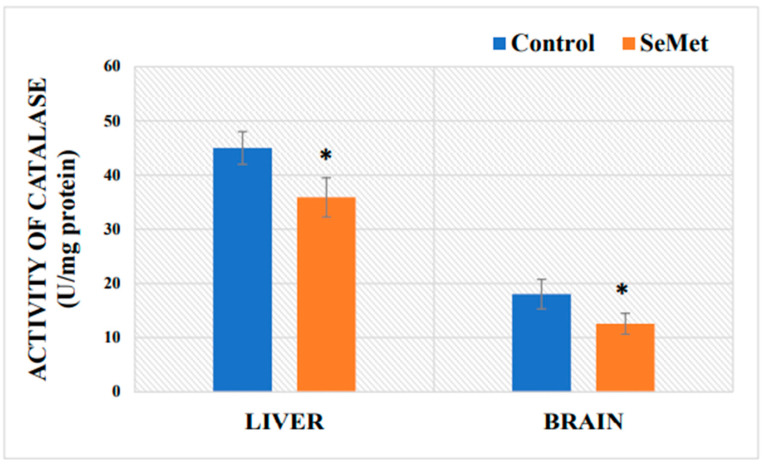
Activity of catalase in mouse brains and livers of control-group mice and mice orally treated with selenomethionine (SeMet) solution for 8 weeks. The model of selenium exposure to mice is described in the Methods section. Data represents the results of 16 separate experiments (8 mice in each group). Results were expressed as the mean ± SEM. *—differences are statistically significant in comparison to the control group; *p* ˂ 0.05.

In order to evaluate how the 8-week long Se consumption affects the cellular antioxidant system, enzymatic catalase activity in the mouse brain and liver was determined, and the results are shown in Figure 4. The obtained results showed that catalase activity in the livers of mice exposed to SeMet was 20% lower than that of control mice and reached 35.89 ± 3.62 U/mg protein, while catalase activity in the livers of control-group mice reached 45.00 ± 3.014 U/mg protein. Catalase activity in the brains of control-group mice was 18.03 ± 2.73 U/mg. Meanwhile, the activity of this enzyme in the brains of Se-exposed mice was statistically significantly reduced by 30% as compared to that in the controls (12.57 ± 1.93 U/mg).

### 2.5. Determination of mRNA Expression in Mouse Liver and Brain of Antioxidative Proteins after Exposure to Selenomethionine

The amount of Se in the body modulates the expression of genes encoding selenoproteins, which can vary from organ to organ. Therefore, one of our tasks was to evaluate the gene expression of a protein that is directly related to the regulation of Se homeostasis. For that purpose, selenoprotein P, which not only has antioxidant properties but is directly involved in Se transport and accumulation (encoded by the *SelenoP* gene), was chosen. Se is also known to influence the expression of non-selenoproteins genes. Therefore, we sought to evaluate whether the excess Se affects the gene expression of catalase and superoxide dismutase—antioxidant enzymes of the cell. These enzymes directly participate in antioxidant defense: SOD by dismutation of the superoxide radical into molecular oxygen and hydrogen peroxide, catalase by breaking down hydrogen peroxide to oxygen and water. Neither superoxide dismutase nor catalase directly depend on Se as a cofactor.

**Figure 5:**
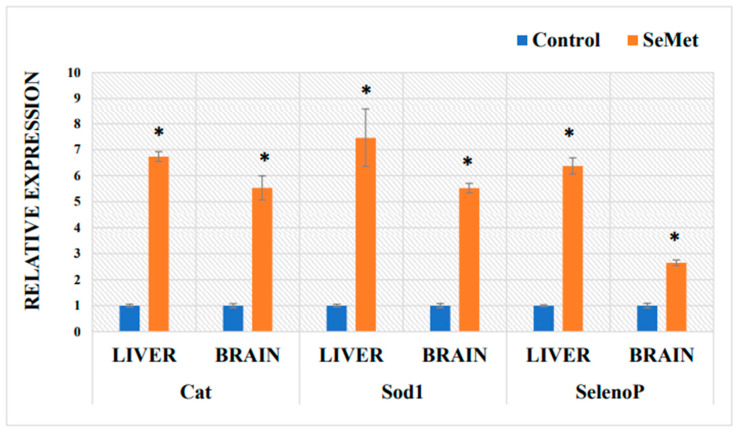
Quantitative real-time PCR validation of transcript expression in mouse brains and livers of control-group mice and mice orally treated with selenomethionine solution for 8 weeks. The relative expression of the target gene was normalized against GAPDH. The model of selenium exposure to mice is described in the Methods section. Data represents the results of 16 separate experiments (8 mice in each group). Results are expressed as the mean ± SEM. *—differences are statistically significant in comparison to the control group; *p* ˂ 0.01.

The mRNA expression of *Cat*, *Sod1*, and *SelenoP* from mouse liver and brain tissue was quantified using RT-qPCR and normalized to the endogenous control GAPDH. The data that represents the effects of Se on antioxidant enzymes and selenoprotein P mRNA expression in mouse organs are represented in Figure 5. *Cat* and *Sod1* genes of control mice were highly expressed in the liver, while the mRNA level of *SelenoP* was lower. Treatment with SeMet (0.4 mg Se/kg BW) elicited the significant up-regulation of mRNA expression of catalase, superoxide dismutase 1, and selenoprotein P in the mouse liver. Under the same conditions, there were significant increases in the levels of brain mRNA encoding *Cat*, *Sod1*, and *SelenoP* in the Se-exposure group. In the liver, the increase in the expression of all studied genes was higher compared to that in the brain.

## 3. Discussion

Due to the dual antioxidant–prooxidant properties of Se, data on how effectively, yet safely, to use Se for both prophylactic and therapeutic purposes are still lacking, while the existing data are often quite conflicting. An optimal amount of Se in the diet ensures the proper synthesis of selenoproteins, preventing the onset of many diseases, such as cancer, neurodegenerative, cardiovascular diseases, and fertility disorders. Although high doses of Se are toxic and are believed to increase the risk of endocrine system and mental disorders and cancer, on the other hand, supra-nutritional doses of Se can be employed as chemotherapeutic agents for their pro-oxidant and pro-apoptotic action against cancer cells [53,54]. The data of other researchers suggest that the therapeutic effect of Se is mostly related to the additional consumption of Se and is observed when Se doses are several times higher than those required to prevent clinical signs of Se deficiency; therefore, we were specifically interested in the consequences of excess Se consumption. The choice of Se dose in our experimental study was based on the results of our previous study, where the effects of sodium selenite exposure to mice were evaluated. Thus, in our study, the dose of Se that laboratory mice received daily in the drinking water in the form of selenomethionine was four times (0.4 mg Se/kg BW) higher than the recommended amount of Se for laboratory mice (0.1 mg Se/kg BW) per day [55]. Scientific evidence indicates that organic Se compounds are more readily absorbed and less toxic than the inorganic forms of Se, so selenomethionine was the organic Se of choice in our study. The choice of duration of exposure in our study was also based on literature data, which indicate that the duration of the experiments in order to evaluate sub-chronic effects usually lasts up to 3 months; thus, mice in our experimental study received selenomethionine in the drinking water for the period of 8 weeks. The route of exposure was chosen per os, since Se usually enters the human body with food or in the form of supplements, the use of which often lasts a month or more.

An adequate intake of Se is very important for physiological processes, such as growth, maintenance of the immune system, normal activity of the brain [56], and various other biochemical functions of the body [57]. Se deficiency on the other hand is associated with bone tissue metabolism disturbances [58], thyroid hormone regulation disorders [59] and many others. The evaluation of the overall systemic effect of SeMet in our study was evaluated based on the changes in mouse body weight and relative organ mass index (the ratio of organ mass to body weight), which is a more accurate indicator than organ mass, over the period of 8 weeks. In toxicological studies, body weight and relative organ mass are important and significant criteria for toxicity assessment [52]. In our study, all BALB/c mice survived to the end of the experiment. The daily oral administration of SeMet did not show a positive effect on the growth of laboratory mice during the 2-month experimental period. The opposite results were observed—throughout the whole experiment, SeMet-exposed mice grew more slowly than the control mice; there was also a small but statistically significant decrease in body weight observed at the 4th and 6th weeks of the study. At the end of the experiment, the final body weight gain of SeMet-supplemented mice was 2.2%, while the control mice gained 4.8% of weight as compared to the initial body weight; however, these differences were not statistically significant. We did not find data on the effect of Se excess on the body weight growth in laboratory mice using a similar experimental model. However, our observations were partially confirmed by the results of a study with rats, which showed that rats that received 5 μg of inorganic Se with every gram of feed slowed in their body weight growth starting from the 10th day and continued to slow down until the end of the experiment (day 28) as compared to rats receiving significantly lower amounts of Se (0.08, 0.24, 0.8 μg Se/1 g feed) [60]. The changes in the body weight of mice are also reflected in the reduced relative organ mass indexes. Differences between the organ mass indexes of the SeMet-exposed and control mice were minimal in the liver and kidney, and a statistically significant decrease was only found in the brain. We could not find, however, any comparable results from other scientists who have used the same/similar exposure doses of Se; on the contrary, Zhang et al. reported an increase, observed in the mass indexes of the liver and kidneys of mice exposed to 0.4 mg Se/kg BW and some other doses [61]. It is known that high doses of organic Se can induce histological changes in the internal organs. For example, the long-term use of Se causes hepatocyte degeneration and vacuolization, which then leads to the appearance of necrotic foci and ultrastructural changes in the liver [62,63]. In the further stage of research, Se concentrations were determined in the blood, liver, and brain of BALB/c mice. The obtained results showed that orally administrated Se enters the tissues and is accumulated there. Our results indicate, that long-term SeMet administration increased the concentration of Se in the blood of mice by 12-fold and in the liver by about 15.4-fold, while the amount of Se in the brain was found to be 42.2-fold higher than that of the control mice. Our results are consistent with the data provided by other scientists, which confirm Se accumulation in the liver, as well as an increased Se concentration in the blood of various species [61,64,65,66]. A comparison of our results with the results of our previously published study, when mice were exposed to the same (0.4 mg Se/kg BW) dose of inorganic Se (Na selenite), showed that blood Se levels in mice exposed to SeMet were three times higher compared with its level in sodium selenite-exposed mouse blood [67]. According to our results, the amount of Se accumulated in the liver of control mice reached 0.615 ± 0.095 µg/g tissue; other authors indicate slightly lower, however, comparable amounts of Se (0.44 ± 0.26 µg/g) in the liver of control mice [68]. Eight-week-long mouse exposure to 0.4 mg of SeMet increased the concentration of accumulated Se in the liver up to 9.49 ± 0.33 µg/g, while results of our previous study showed that the concentration of Se accumulated in the liver of mice exposed to the same dose of sodium selenite was 2.11 ± 0.045 µg/g [67], i.e., 4.5 times lower. Determination of Se contents in the brains of mice revealed that the brain accumulates 10 times less Se than the liver; however, the long-term administration of SeMet solution increased brain Se more than 42 times (2.57 ± 0.147 µg/g) compared to that in the control. Meanwhile, after mouse exposure to 0.4 mg of sodium selenite for the same period, the concentration of Se accumulated in the brain was only 2.5 times higher as compared to that in the control [67]. Therefore, a summarization of our results shows that the absorption of organic Se is significantly more efficient than inorganic Se. Our statement agrees with the claim of other authors, confirming that it is organic Se (selenomethionine, selenocysteine) that is the best form of Se absorbed by the body, although other forms of Se are also absorbed quite efficiently [69]. H. P. Blossom with co-authors points out that SeMet is almost completely absorbed (97%), while selenite absorption was lower (57%) [70]. Data of our study on the influence of long-term SeMet consumption on the accumulation of this element in the brain and liver complement the results of other authors. N. Akahoshi with co-authors [71] showed that 6-week-long SeMet-supplemented feed consumption (20 mg SeMet/kg feed) increased the amount of Se in both the livers and the brains of mice. The highest amounts of Se were accumulated in the liver (~9 μg/g); however, the significantly increased Se was also found in the brain (~2.5 μg/g). Unfortunately, it should be emphasized that it is rather difficult to accurately compare the data of different studies on the amounts of Se accumulated in the tissues; data are relatively scarce, often quite contradictory, and differences in experimental animal models must also be considered.

Selenium levels have been shown to modulate selenoprotein expression in mammals and cells in vitro. This regulation mostly occurs at the translation, and moderately at the mRNA transcription, level. This type of selective regulation of expression ensures that the synthesis of essential selenoproteins is maintained at the expense of others [3,72]. It was shown that the GPx level is increased in animals treated with an excess of Se [73]. mRNA levels and GPx activity were significantly increased in rats injected with 20 mg Se/kg per day but decreased after the injection of 40 or 80 mg Se/kg per day [74]. Other authors indicate that the GPx4 mRNA level in chickens was downregulated by an excess of Se [75]. When dietary Se was increased from 0.3 to 3.0 mg Se/kg, testicular mRNA levels of Txnrd1 and selenoprotein 15 (Sep15) were attenuated, whereas the expression of Gpx1 was increased in the pig liver [76]. A ten-fold increase in dietary Se (from 0.3 to 3.0 mg Se/kg) decreased testicular mRNA levels of Txnrd1 and selenoprotein 15 (Sep15), whereas *Gpx1* expression in the pig liver was found to be increased [76]. Excess Se can modulate the gene expression of selenoproteins in various organs; however, the liver is the main one to regulate whole-body Se levels; it synthesizes the most selenoproteins, which then reach extrahepatic tissues and maintain normal Se concentrations in different organs and cells [3]; one such key proteins involved in Se transport is the selenoprotein P [77] Although it has been shown that in the case of excess Se, the liver increases its removal from the body, thus ensuring a stable level of Se in the tissues [3], the results of our study show that after the long-term consumption of SeMet, the brain, not the liver, accumulated the highest amount of Se as compared to the controls. Scientific data show that although the majority of selenoprotein P is synthesized by liver cells, the expression of the selenoprotein P gene also increases in the brain [78]; this is confirmed by the results of our study, which showed that with a significant increase in the amount of Se in the blood, the expression of the *SelenoP* gene significantly increased both in the liver and brain. It is likely that with a significant excess of Se, the liver itself is unable to ensure the Se balance in the body, so the brain is also involved in maintaining the homeostasis of this element, since selenoprotein P is the most important protein that regulates the amount of Se in nervous tissue [78]. Z-H. Zhang and G-L. Song [79] found 24 selenoprotein-encoding genes that are expressed in the mouse brain. Genes encoding selenoproteins, such as Sep15, SelM, SelK, SelP, GPx4, and SelW, have higher expression levels than other selenoproteins, while *SelenoP* gene expression is active in more than 90% of the areas of the brain. There are data suggesting that the amounts of Se in the brain and cerebrospinal fluid do not actually depend on the amount of Se in the blood, since the brain, being a priority organ, is constantly protected from the deficiency of this trace element [12]. However, in the case of excess Se, in order to avoid its toxicity, Se entry into the nervous tissue should be strictly regulated by the blood–brain barrier [80]. The increase in Se levels in different mouse tissues could possibly be viewed positively if we assume that this is related to the tissue’s ability to take advantage of Se’s antioxidant properties. However, in order to make sure that high amounts of Se accumulated in tissues do not cause cellular oxidative stress, we evaluated the effect of SeMet on the formation of malondialdehyde, an important marker—the end product of polyunsaturated fatty acid peroxidation—and gene expression of the first line of antioxidant defense enzymes, such as superoxide dismutase *Sod1* and catalase *Cat* [30]. The results obtained in our study showed that the selected dose of Se was high enough to increase the formation of reactive oxygen species (ROS) and activate lipid peroxidation in the brain and blood after 8 weeks. Despite the fact that erythrocytes are resistant to oxidation and blood acts as an effective systemic redox buffer, the greatest change in MDA content was observed in the blood of mice [81]. On the other hand, erythrocytes are rich in oxidants, such as oxygen or iron, which, if released, become a great catalyst for ROS formation during the Fenton reaction. The high concentration of polyunsaturated fatty acids in the erythrocyte membrane (40%) makes them susceptible to peroxidation, which results in the loss of erythrocyte membrane integrity and reduced membrane enzyme activity [81]. Therefore, as a result of lipid peroxidation, an increased level of MDA was observed in the blood. In our study, an increased lipid peroxidation was also observed in the brain, which confirms the oxidative stress in the nervous tissue cells. The MDA increase in the brains of mice may be related to nerve tissue functions and structure. Due to the high concentration of polyunsaturated fatty acids, the brain is particularly susceptible to free radical damage and oxidative stress. Fatty acids are part of the complex lipids, which make up the membranes of neurons [82]. Nervous tissue requires a large amount of energy and O_2_ to function properly [83]. Literature data indicate that high doses of Se can have a toxic effect and increase the formation of ROS [29,84]. Selenium can negatively affect the redox state of cells and cause cell apoptosis, either directly oxidizing protein thiol groups and glutathione cysteine residues, or indirectly increasing the formation of ROS [18]. As the amount of Se in the cells increases, it reacts with reduced glutathione (GSH) to form highly active selenopersulfide (GSSe-), which further reacts with a new GSH molecule to form superoxide anion [85]. The pro-oxidant effect of Se is mostly related to its inorganic forms, whereas selenomethionine and selenocysteine are known to be less toxic selenium-compounds [86]. The results of our study are not sufficient in order to state that the pro-oxidant effect of Se in the mouse brain is direct. That might be related to the disturbance of homeostasis of other bioelements. Our results showed that long-term exposure to Se increases the levels of iron, copper, and zinc in the brains of BALB/c mice. The tight regulation of the amount of these biogenic elements is very important for maintaining normal brain activity, as they play an important role not only in brain physiology, but also in pathophysiology [87]. The role of Cu, Zn, and Fe is particularly important for the development of neurodegenerative diseases, as these metals can affect protein structure (misfolding) and the occurrence of oxidative stress [45]. Iron (in excess) participates in Fenton and Haber–Weiss chemistry and generates significant amounts of toxic hydroxyl radicals, which promote lipid peroxidation and cell death (ferroptosis) [88]. The generation of ROS, which is directly involved in the inflammatory process, can significantly affect iron metabolism via their interaction with iron-regulatory proteins (IRPs). The pro-inflammatory cytokines induce changes in the iron proteins responsible for maintaining iron homeostasis, such that increased amounts of iron will be deposited in cells in the brain [89]. Excess iron in the brain causes neurotoxicity and significant cognitive impairments, which has been implicated in the pathogenesis of several neurological disorders, including hypoxic ischemic brain injury and periventricular white matter injury in neonates [90], as well as neurodegenerative disorders in elders [91,92]. Excessive iron-dependent cell death—ferroptosis—is usually accompanied by lipid peroxidation and is closely related to the pathophysiological processes of many diseases [93]. Accordingly, iron homeostasis must be tightly controlled [94].

Iron-induced damage due to the increased ROS generation in the brain is thought to be related to a lower ROS tolerance of nervous tissue and a weaker response of the antioxidant system [95] than, for example, erythrocytes or the liver [96], where, according to our results, the expression of the genes encoding the first-line defense antioxidant enzymes *Sod1* and *Cat*, as well as the gene encoding selenoprotein P, *SelenoP*, was increased. There is scientific data indicating that selenoprotein P, in addition to participating in the maintenance of Se homeostasis in the body, also exhibits metal-binding and glutathione peroxidase-like activities [24,97]. Although an evaluation of gene expression of antioxidant system enzymes in the blood was not performed, it is likely that increased erythrocyte catalase activity and decreased iron and copper levels (as compared to those in the control group) could reduce the level of oxidative stress in the blood under long-term exposure to SeMet.

Zinc and copper ions are required for proper brain antioxidant protection [40,41]. Zinc acts as an antioxidant, and it protects protein sulfhydryl groups from oxidation and increases the expression of metallothionein [98]. Thus, in circumstances when the cells are deficient of this trace element, or, on the contrary, its excess occurs, Zn becomes a proinflammatory and proapoptotic factor and cells experience a condition known as oxidative stress [98,99]. Zinc deficiency or excess is neurotoxic and is involved in the pathogenesis of neurological diseases, such as amyotrophic lateral sclerosis, depression, schizophrenia, Parkinson’s disease, and Alzheimer’s disease [100,101,102]. The inclusion of copper in the composition of metallothionein is one of the examples of cell defense mechanisms to protect cell structures from Cu toxicity and prevent oxidative damage [103,104]. Metallothioneins, low-molecular-weight cysteine-rich proteins expressed in astrocytes and neurons, strictly regulate the amount of free Cu in the cell in order to avoid the toxic formation of hydroxyl radicals [105]. There is evidence that impaired copper homeostasis in Alzheimer’s patients leads to oxidative stress and neurodegeneration [106], resulting in memory impairment. Copper homeostasis in the brain must be strictly regulated, because due to its similar redox behavior to iron, it participates in Haber–Weiss and Fenton reactions, thus causing oxidative stress in nervous cells. The disruption of copper and zinc homoeostasis results in an excess of ROS, which then causes DNA damage, protein modifications, and possibly cancer development [107]. Zinc and copper deficiencies or excess in the developing brain are considered risk factors for autism-like disorders [108,109,110]. Excess zinc can lead to copper deficiency, which is associated with a number of negative effects, such as reduced expression of the antioxidant defense system enzyme superoxide dismutase [99,111]. Results of our study show an increase in both Zn and Cu concentrations in the brain, as well as increased expression of the *Sod1* gene. Superoxide dismutase is the first enzyme involved in ROS neutralization, catalyzing the dismutation of two superoxide anion molecules into H_2_O_2_ and O_2_ it neutralizes the harmful superoxide anion. SOD is a metalloenzyme that requires a metal cofactor (Fe, Zn, Cu, or Mn) to function [107,112,113]. Fe-SOD is mostly found in prokaryotes and chloroplasts of some plants, and Mn-SOD is found in prokaryotes and eukaryotic mitochondria, while Cu/Zn-SOD is mainly found in the cytosol of eukaryotes but also found in chloroplasts and peroxisomes. Humans and other mammals have three forms of SOD: SOD1 is found in the cytoplasm, SOD2 is found in the mitochondria, and SOD3 is found in the extracellular medium. SOD1 is a dimer, while SOD2 and SOD3 are tetramers. SOD1 and SOD3 contain Cu and Zn in their active centers, and SOD2 contains Mn. Coding genes are located on chromosomes 21, 6, and 4 (21q22.1, 6q25.3, and 4p15.3-p15.1), respectively [114]. The increase in *Sod1* gene expression in the brain found in our study suggests that the formation of ROS in the nervous tissue was indeed increased after 8 weeks of 0.4 mg Se/kg BW per os consumption. H_2_O_2_ formed in the superoxide dismutase-catalyzed reaction, although not a radical, can oxidize protein -SH groups and cofactor-metal ions or release iron from Fe-S centers. The results of our study showed an increase in the Fe content both in the brain and in the liver of BALB/c mice. Therefore, it is important to emphasize the potential role of iron in the mechanism of Se pro-oxidant action. In the case of an Fe excess, through Fenton or Haber–Weiss reactions, H_2_O_2_ is converted into a hydroxyl radical, which not only damages proteins, DNA, and lipids [115], but can also inhibit the regulation or synthesis of antioxidant enzymes [116], even if the expression of the gene itself is increased. This was shown by the results of our study—catalase activity was decreased in both the brain and liver, suggesting that this may be a consequence of ROS exposure to proteins, including the enzyme itself. The effect of ROS can be threefold: oxidation of amino acid residues, disturbance of the peptide bonds, or aggregation of the proteins [117]. Catalase is an enzyme of the antioxidant system that breaks down hydrogen peroxide into water and molecular oxygen. It is a tetrameric holoenzyme with one heme in each subunit. Two of the subunits can each bind a molecule of NADPH. The enzymatic activity of catalase requires Fe or Mn as a cofactor [118]. The human *CAT* gene is located on chromosome 11 (11p13). Catalase has two types of activity: catalytic (decomposes H_2_O_2_ into H_2_O and O_2_) and peroxidic (oxidizes low-molecular-weight alcohols in the presence of low H_2_O_2_ concentrations). It is a very efficient enzyme that can break down millions of H_2_O_2_ molecules per second. Catalase is mainly found in peroxisomes and is also present in the cytoplasm (e.g., erythrocytes) and nucleus, but is absent in the mitochondria of mammalian cells [119], where H_2_O_2_ is broken down by another enzyme—glutathione peroxidase. It is believed that catalase breaks down only part of H_2_O_2_ molecules, the other part participates in physiological processes, such as signal transmission during cell proliferation, cell apoptosis, platelet activation, and the maintenance of a normal redox balance [120]. However, high concentrations of H_2_O_2_ are very harmful to cells [121]. It is known that catalase activity is inhibited by various compounds, including its natural substrate H_2_O_2_ (at a concentration higher than 0.1 mol) [122], or excess MDA [30]. Thus, there could have been several reasons leading to the inhibition of catalase activity in the organs we studied. Thus, although an increase in *Cat* gene mRNA levels may be observed under SeMet exposure, this may not necessarily be reflected in the amount of active protein. Various molecular mechanisms, such as miRNAs, lncRNAs, and others, can interfere with efficient protein translation, so additional studies are needed to better understand the influence of selenomethionine. Results of our study showed a decrease in the level of MDA found in the liver of experimental mice, which suggests that the excess of SeMet was not toxic to this organ. On the contrary SeMet seemed to activate the antioxidant system of hepatocytes, in this way improving the redox status of cells and preventing lipids from peroxidation, thereby reducing MDA levels [123]. These findings agree with the results of the study with Wistar rats that were fed a Se-supplemented diet wherein a decrease in hepatic MDA was also observed [124]. Active antioxidant defense is also confirmed by the increase in the expression of *Cat* and *Sod1* genes in the liver. However, it should be noted that although there is a significant increase in the amount of mRNA in the brain tissue, the gene expression remains significantly lower compared to that in the liver tissue.

A summary of the obtained results showed that the liver of control BALB/c mice contains more Se than the brain. After 8-week-long selenomethionine-supplemented water consumption, the level of selenium in the blood was elevated by 12-fold, in the liver, it was elevated 15-fold, and in the brain, it was elevated 42-fold, as compared to levels in the control group of mice. Long-term selenomethionine administration caused disturbances in the homeostasis of iron, zinc, and copper: blood amounts of Fe and Cu were decreased; however, liver amounts of Fe and Zn, as well as brain levels of all examined elements, were increased. In the brain and blood, selenomethionine acted as a pro-oxidant by statistically significantly increasing the level of malondialdehyde, while in the liver, on the contrary, it decreased the level of lipid peroxidation. In our experimental conditions, Se changed both the expression of selenoprotein P, which is involved in selenium homeostasis, and Se-independent enzymes—superoxide dismutase and catalase expression. The mRNA expression level of all of these proteins was increased in the brain, and especially in the liver, in response to selenomethionine exposure. Meanwhile, the enzymatic activity of catalase was inhibited in both examined organs.

## 4. Materials and Methods

### 4.1. Chemicals and Reagents

All reagents used in the experimental activities were of analytical grade. Tris, Na_2_SeO_3_, H_3_PO_4_, KCl, n-butanol, HCl, and 2-mercaptoethanol were purchased from Sigma-Aldrich (Steinheim, Germany); H_2_O_2_, HNO_3_, MgCl_2_, TBA (thiobarbituric acid), and sucrose were acquired from Serva (Heidelberg, Germany); Maxima SYBR Green/ROX qPCR Master Mix (2X), “High Capacity cDNA Reverse Transcription kit”, and the “Gene JET RNA purification kit” were obtained from Thermo Fisher Scientific (Waltham, MA, USA), Standard reference material 1577c was obtained from NIST (Gaithersburg, MD, USA).

### 4.2. Animals and Experimental Procedure

Animal care and procedures were carried out in accordance with the rules established by the European Convention on the Protection of Vertebrate Animals Used for Experimental and Other Scientific Purposes (License of the State Veterinary Service of the Republic of Lithuania to work with laboratory animals No. G2-203). Here, 4–6-week-old white BALB/c female laboratory mice with an initial body weight of 20–25 g were used as experimental subjects. Mice were randomly divided into two groups of 8 animals. Mice of the first control group had free access to tap water, while animals of the second group were given ad libitum tap water supplemented with selenomethionine (0.4 mg Se/kg body weight (BW)) for the period of 8 weeks. To evaluate the changes in the body weight, mice were weighed once a week. After 8 weeks, the animals of each group were anesthetized and terminated.

### 4.3. Determination of Malondialdehyde Content

To determine the extent of lipid peroxidation in biological samples, the content of malondialdehyde (MDA) was measured. MDA forms as a result of a reaction with TBA and is expressed in nmol/g of wet tissue weight. The organs of mice were homogenized with 9 volumes *w/v* of cold 1.15% KCl to obtain a 10% homogenate. Then, 0.5 mL of the homogenate was mixed with 3 mL 1% H_3_PO_4_ and 1 mL 0.6% TBA aqueous solution. The reaction mixture was heated for 45 min in a boiling water bath, and after cooling, 4 mL of n-butanol was added and mixed thoroughly. The butanol phase was separated by centrifugation (Beckman J2-21, Beckman Instruments, Palo Alto, CA, USA) and used to determine light absorbance (UV/Vis spectrophotometer LAMBDA 25, (Perkin Elmer, Waltham, MA, USA) at 535 and 520 nm [125].

### 4.4. Determination of Trace Element Concentrations

The contents of Se, Fe, Zn, and Cu in the blood, brain, and liver of BALB/c mice were established using an inductively coupled plasma mass spectrometer NexION 300 D (Perkin Elmer, Waltham, MA, USA) after microwave-assisted acid digestion. For acid digestion, samples consisting of 1 mL 69% HNO_3_, 1 mL 32% H_2_O_2_, 100 mg of biological sample, and 5 mL H_2_O were prepared in glassware, which was placed in a microwave system Anton Paar Multiwave 3000 (Graz, Austria). After digestion, samples were diluted with deionized ultrapure water; the obtained solution was used for elemental analysis with an ICP-MS system. To ensure analytical accuracy, internal and external quality control procedures were conducted, and the control of laboratory equipment contamination with trace elements was performed.

Method detection limits (MDLs) and quantifications limits (LOQs) were calculated from replicated measurements of the blanks. For Cu µg/L, n = 10, mean ± SD—0.18 ± 0.04; LOD—0.038; LOQ—0.13. For Zn µg/L, n = 7, mean ± SD—0.59 ± 0.11; LOD—0.14; LOQ—0.45. For Se µg/L, n = 7, mean ± SD—0.002 ± 0.0013; LOD—0.0006; LOQ—0.002. For Fe µg/L, n = 7, mean ± SD—1.15 ± 0.30; LOD—0.39; LOQ—1.33. For the blood analysis ClinChek^®®^ Whole Blood Control for Trace Elements, Level I was used. The mean ± SD and variation coefficients (%) were calculated: Cu mg/L—0.70 ± 10.98, 1.6%; Zn mg/L—4.91 ± 0.36, 7.3%; Se µg/L—73.61 ± 7.61, 10.3%; Fe mg/L—365.24 ± 68.53, 18.8%. For organ analysis standard reference material of bovine liver was used. The mean ± SD and variation coefficients (%) were calculated: Cu µg/g—276.10 ± 24.82, 8.7%; Zn µg/g—181.35 ± 18.95, 7.3%; Se µg/g—2.04 ± 0.15, 7.3%; Fe µg/g—198.52 ± 10.84, 5.5%.

The blood concentration of Se was expressed in µg/L; meanwhile, concentrations of Fe, Zn, and Cu were expressed in mg/L. Tissue concentrations of Se, Fe, Zn, and Cu were expressed in µg/g.

### 4.5. Preparation of the Brain and Liver Homogenates

After cervical dislocation, the brain and liver were removed and immediately placed on ice. After weighing, organs were homogenized in three volumes (relative to organ weight) of buffer (pH 7.6), which was prepared using 50 mM Tris-HCl, 250 mM sucrose, 60 mM KCl, 5 mM MgCl_2_, and 10 mM 2-mercaptoethanol. The obtained homogenate was centrifuged at 15,000× *g* for 15 min with a centrifuge Beckman J2-21 (Beckman Instruments, Palo Alto, CA, USA), and resulting postmitochondrial supernatant was used for the measurement of enzymatic activity in the tissue.

### 4.6. Protein Concentration Assay

The content of protein in homogenate samples of the brain and liver was determined according to Lowry et al. [126].

### 4.7. Measurement of Enzyme Catalase Activity

Enzymatic activity of tissue catalase (CAT) was assessed according to the H_2_O_2_ reaction with ammonium molybdate [127], in which a yellowish complex that absorbs at a 410 nm light wavelength was produced. Enzymatic activity was expressed in U/mg protein. One unit of catalase (U) decomposes 1 μmol of H_2_O_2_ per minute.

### 4.8. RNA Extraction and Real-Time Quantitative Reverse Transcription (RT) PCR (RT-qPCR)

Total RNA was extracted from mouse brain and liver samples using a GeneJET RNA Purification Kit (cat.no. K0731, Thermo Fisher Scientific, Waltham, MA, USA). The quantity and quality of extracted RNA were assessed using a NanoDrop spectrophotometer (Thermo Scientific, Wilmington, DE, USA).

Total RNA extracted from the liver was eluted in 50 µL and that extracted from the brain was eluted in 25 µL of nuclease-free water (RNA yield ranged from 500 to 4000 ng/µL in the liver and from 200 to 1000 ng/µL in the brain). The absorption ratio of OD260 nm/OD280 nm was between 1.8 and 2.2.

The mRNA levels of superoxide dismutase, catalase, and selenoprotein P in mouse brain and liver tissue were assessed via quantitative real time RT-PCR. For this, 1 µg of total RNA from each sample was reverse transcribed in a total volume of 20 µL according to the manufacturer’s instructions (High-Capacity cDNA Reverse Transcription Kit, cat.no. 4374966, Thermo Fisher Scientific, Waltham, MA, USA). Each real-time PCR, the reaction was performed using 3 µL of a 5 ng/µL concentration of cDNA, 0.5 µL of each primer, and 6 µL of Power SYBR Green PCR Master Mix, in a final volume of 12 µL per reaction.

Primers were designed to span an intron within the corresponding genomic sequence using NCBI Primer-BLAST (https://www.ncbi.nlm.nih.gov/tools/primer-blast/accessed on 21 May 2022) and synthesized by Invitrogen. Primer sequences are listed in Table 2.

Cycling parameters for real time PCR were 95 °C for 10 min, 40 cycles of 95 °C for 15 s, 60 °C for 30 s, and 72 °C for 30 s, using a “QuantStudio™ 3 Real-Time PCR System” (cat. no. A28136, Applied Biosystems, Beverly, MA, USA).

The real-time quantitative PCR reaction was performed in triplicate for each sample, and the mean value was used to calculate mRNA expression levels. Six biological replicates were measured for each group.

The fold-change (n-fold) for gene expression was calculated using the relative quantification method (2-ddCt), using GAPDH as the endogenous control. The average dCt for control group brain and liver samples collected without drinking selenomethionine were used as the calibrator for each sample.

Briefly, using SOD1 as an example: dCt normalized target = SOD1 Ct − GAPDH Ct; ddCt = dCt normalized target − dCt normalized calibrator (control group); and then, the n-fold (2-ddCt) could be calculated. Therefore, the n-fold represented the gene expression for each sample in each mouse in relation to that in samples from the control group, normalized to the endogenous control GAPDH.

### 4.9. Statistical Analysis

Statistical analysis was performed using the statistical software package IBM SPSS Statistics (1.0.0.1416 version). Results are expressed as the mean ± standard error of the mean (SEM). The data were analyzed based on a nonparametric Kruskal–Wallis test. The statistical analyses of mRNA expression were performed on the delta delta Ct (ddCt) values and then converted to n-fold (2-ddCt) for data presentation. Statistical significance was set at *p* ˂ 0.05. Graphical analysis of the results was performed with the MS Excel (2019) computer program.

## 5. Conclusions

After the summarization of our results, one can conclude that after 2-month-long exposure, Se has acted as a pro-oxidant in the brain, and its action was possibly enhanced by increased levels of Fe, Cu, and Zn. It appears that the brain was unable to protect itself from the adverse effects of excess Se. Although Sod1, Cat, and especially *SelenoP* gene expression was active in the brain, it was not as active as compared to that in the liver. In the liver, however, a decrease in the lipid peroxidation and active expression of *Sod1*, *Cat*, and especially *SelenoP* genes were observed, indicating that the antioxidant protection of this organ, despite the disturbed Fe and Zn homeostasis, has helped the cells to avoid oxidative stress conditions. Although one of the most important functions of the liver is to detoxify harmful compounds and ROS in order to protect, from their toxicity, not only hepatic but other tissues as well, it is possible that the capacity of the liver’s protective systems to defend the brain was insufficient, or it was exhausted over a long period of time. In the future, more detailed studies of the cellular antioxidant system are needed to help to answer unanswered questions.

## Figures and Tables

**Table 1:** Concentrations of selenium, iron, copper, and zinc in the blood, brain, and liver of the control group of mice and mice orally treated with selenomethionine (SeMet) solution for 8 weeks. The model of selenium exposure to mice is described in the Methods section. Data represents the results of eight separate experiments. Results are expressed as the mean ± SEM. *—differences are statistically significant in comparison to the control group; *p* ˂ 0.05.

Mouse Group	Trace Element	Blood (µg/L Se) (mg/L Fe, Cu, Zn)	Brain (µg/g)	Liver (µg/g)
Control	Selenium (Se)	210.508 ± 24.138	0.061 ± 0.012	0.615 ± 0.095
SeMet		2526.303 ± 181.058 *	2.573 ± 0.147 *	9.490 ± 0.331 *
Control	Iron (Fe)	706.097 ± 32.350	27.265 ± 1.895	119.068 ± 11.259
SeMet		497.121 ± 9.849 *	38.803 ± 2.157 *	272.894 ± 9.521 *
Control	Copper (Cu)	0.985 ± 0.064	3.715 ± 0.164	7.795 ± 0.416
SeMet		0.594 ± 0.026 *	6.266 ± 0.581 *	6.877 ±0.125 *
Control	Zinc (Zn)	6.640 ± 0.294	16.863 ± 0.505	32.453 ± 1.403
SeMet		6.307 ± 0.219	24.624 ± 2.389 *	24.624 ± 2.389 *

**Table 2:** Primer sequences.

Genes	Forward Primer, 5′-3′	Reverse Primer, 5′-3′
*Sod1*	AGCATGGCGATGAAAGCGG	CCTGCACTGGTACAGCCTTGT
*Cat*	AAGATTGCCTTCTCCGGGTG	GACATCAGGTCTCTGCGAGG
*SelenoP*	GAAACTGTTCAGGGGCTTGC	CACATTGCTGAGGTTGTCCTCG

## Data Availability

Data is contained within the article.
